# An Optimized Framework for Breast Cancer Classification Using Machine Learning

**DOI:** 10.1155/2022/8482022

**Published:** 2022-02-18

**Authors:** Epimack Michael, He Ma, Hong Li, Shouliang Qi

**Affiliations:** College of Medicine and Biological Information Engineering, Northeastern University, Shenyang 110169, China

## Abstract

Breast cancer, if diagnosed and treated early, has a better chance of surviving. Many studies have shown that a larger number of ultrasound images are generated every day, and the number of radiologists able to analyze this medical data is very limited. This often results in misclassification of breast lesions, resulting in a high false-positive rate. In this article, we propose a computer-aided diagnosis (CAD) system that can automatically generate an optimized algorithm. To train machine learning, we employ 13 features out of 185 available. Five machine learning classifiers were used to classify malignant versus benign tumors. The experimental results revealed Bayesian optimization with a tree-structured Parzen estimator based on a machine learning classifier for 10-fold cross-validation. The LightGBM classifier performs better than the other four classifiers, achieving 99.86% accuracy, 100.0% precision, 99.60% recall, and 99.80% for the FI score.

## 1. Introduction

Breast cancer represents one of the primary diseases behind the loss of life for women globally. It can be classified into three (3) groups: normal, benign, and malignant tumors. Besides, it is grouped into five (5) stages (0-IV). However, these stages are distinguished by the size of tumors, identified as invasive or noninvasive cancer, which have lymph nodes and spread to other parts, although the chances of survival decrease as the cancer progresses to stage IV [1]. Consequently, early detection and analysis of breast cancer increase the probability of survival and decrease the mortality rate. As reported by the American Cancer Society, it is estimated that 327,610 cases will be diagnosed in 2020, which comprises 276,480 invasive breast cancers for women, 2620 for men, and 48,530 cases of ductal carcinoma in situ identified in women. There are approximately 42,690 people expected to die in 2020, including 42,690 women and 520 men [2]. In contrast, the World Health Organization (WHO) reported that breast cancer was the most diagnosed disease among women internationally. It is estimated that 627,000 women die every year due to breast cancer, and most of this disease occurs in low- and middle-income countries. More than 2.1 million new cases were diagnosed in 2018, and one out of eight women will be diagnosed with invasive breast cancer in their lifetime [3]. In addition, various imaging techniques have been developed and are widely used to detect early breast cancer. Mammography, breast ultrasound, magnetic resonance imaging (MRI), positron emission tomography (PET), and computed tomography (CT) are some imaging techniques [4–6]. Breast ultrasound can be divided into diagnostic and therapeutic categories. Diagnostic ultrasound was considered noninvasive, and therapeutic ultrasound did not produce images [7]. ML for breast cancer classification has been presented in this study. The study shows that, in recent years, the researchers employ metaheuristics such as the ones described below.

### 1.1. Harris Hawks Optimization (HHO)

The HHO is based on a gradient-free optimization technique that can be applied to solve any problem based on optimization [8–11].

### 1.2. Monarch Butterfly Optimization (MBO)

The MBO algorithm is a type of swarm intelligence metaheuristic algorithm inspired by monarch butterfly migration behavior [12]. The method can be used for medical image segmentation [13, 14], feature selection [15–17], and classification [18, 19].

### 1.3. Slime Mould Algorithm (SMA)

The SMA is a population-based metaheuristic algorithm inspired by the phenomenon of slime mould oscillation [20], for segmentation [21, 22], feature selection [23, 24], and classification [25].

### 1.4. Moth Search Algorithm (MSA)

The MSA is a recent swarm intelligent optimization algorithm that mimics moth movement. The method can be used in image segmentation. [26–28], feature selection [29–31], and classification [32, 33].

### 1.5. Hunger Games Search (HGS)

The HGS incorporates the idea of hunger into the feature selection process. The method can be used in segmentation [34, 35], feature selection [36], and classification [37].

### 1.6. Colony Predation Algorithm (CPA)

The CPA method is based on animal corporate predation in nature [38, 39].

However, the majority of machine learning models used to classify breast cancer have had their hyperparameters left with default values, and some have been manually adjusted. In this paper, we proposed automatic ML that can automatically build optimized ML algorithms. The proposed method significantly reduces human labour and can be easily applied in real applications. Contributions to our article are presented below:

- First, it developed an algorithm for detecting and segmenting outliers
- Second, it is developing an algorithm for feature extraction based on the pyradimics toolbox
- Third, we have discussed machine learning models and hyperparameter optimization techniques
- Four, it summarizes the hyperparameter optimization used in each related work
- Five, it identifies and recommends the most effective classifier for breast cancer classification
- Finally, we have evaluated the effect of Bayesian optimization with a tree-structured Parzen estimator

The rest of this work is presented on paper as follows: Section 2 presents the literature of related works.  Section 3 introduces the application of hyperparameter optimization-based machine learning models.  Section 4 provides a general description of the materials and the methods used.  Section 5 describes the experiment setup and testing process. The experiment results are discussed in Section 6. Finally, this study is concluded in Section 7.

## 2. Related Work

This section discusses the previous research on breast cancer classification based on ultrasound image, and Table 1 summarizes each paper based on HPO, which is organized from 2019 to 2021.

Zeebaree et al. [40] developed a CAD that uses ML and region-growing segmentation based on the morphological characteristics of breast ultrasound. The method uses a hybrid model to extract features from the ROI. The features include 7 moments, FD, and HOG instead of one feature. 250 ultrasound images were used, of which 100 were benign and 150 malignant lesions. The ANN is used to classify ultrasound images, and it achieved an accuracy of 93.1% for malignant lesions and 90.4% for benign lesions.

Adel et al. [41] proposed a method for detecting and classifying breast cancer using B-mode and elastography images. The proposed method employs a total of 82 ultrasound images; 56 were malignant lesions, and 26 were benign lesions. The extracted features from ROI were based on geometrical and texture features. The 33 features extracted from B-mode and elastography images include mean, standard deviation, area, perimeter, width-to-height ratio, contrast-to-noise ratio, and signal-to-noise ratio. The SVM was employed and achieved an accuracy of 94.12%.

El-Azizy et al. [42] developed the CAD based on three morphological features extracted from conventional B-mode ultrasound images. The SVM classifier employs features extracted from segment images to classify as benign versus malignant. The features extracted include the perimeter, regularity variance, and circularity range ratio. The CAD developed 323 ultrasound breast images of which 216 were benign lesions and 107 malignant lesions, and the noise was removed using an anisotropic filter. The CAD employs a semiautomatic and automatic approach. The proposed method achieved 95.98% of accuracy, 97.20% of sensitivity, and 95.37% of specificity when the semiautomatic method was applied. Besides, the method achieved 95.67% of accuracy and 95.83% of specificity and decreased sensitivity to 95.33% when employing full automatic segmentation.

González-Luna et al. [43] proposed the CAD for the classification of breast ultrasound images as benign or malignant lesions. The method employs a total number of 2032 ultrasound images of which 1341 were benign and 691 malignant lesions, acquired from the National Cancer Institute (INCa) of Rio de Janeiro, Brazil. The SVM, Ada, LDA, *k*-NN, RBFN, RF, and MLR were applied to classify 137 textures and morphological features. Some features include area difference with an equivalent ellipse, maximum proportional, solidity, extent, roundness, shape class, Pearson's correlation coefficient, and mean squared error. The proposed method shows that LDA outperformed other classifiers as it achieved 89.00% accuracy, 82.00% sensitivity, and 93.00% specificity, and the AUC was observed to be 95.00%.

Wei et al. [44] proposed an automatic classification of breast cancer based on breast ultrasound images. The proposed method uses texture and morphological features to classify images as benign or malignant lesions. The proposed method uses a total of 1061 ultrasound images, including 472 benign and 589 malignant tumors. These features extracted include the direct least-squares fitting of ellipses, compactness, and radial range spectrum extracted from ROI. The SVM classifier was applied to classify morphological features. The results, based on morphological features, yielded 75.94% accuracy, 66.37% sensitivity, 86.87% specificity, and 85.23% precision.

Karwat et al. [45] developed the CAD based on the shape parameter of the Nakagami distribution and GLCM matrix using quantitative ultrasound. A total of 116 ultrasound images were used, of which 57 were malignant lesions and 59 benign lesions. These images were acquired from the Department of Radiology, Maria Skłodowska-Curie Memorial Institute of Oncology in Warsaw. The LDA classifier with cross-validation based on leave-one-out was adopted to classify malignant lesions versus benign lesions. The proposed method produced a classification accuracy of 89.00%.

Rani and Dhenakaran [46] proposed the CAD for the classification of ultrasound breast images based on a modified neural network (MNN) to predict tumor growth rate. The proposed CAD employs 840 ultrasound images of which 270 are malignant lesions and 570 benign images lesions, and a Wiener filter is used to reduce speckle noise. The different attributes include shape, regular (benign) and irregular (malignant), concavity, tumor area, variance, Euclidean distance, standard deviation, and entropy which were extracted based on the foreground and background, after segmentation. The proposed method achieved 97.80% accuracy.

Li et al. [47] developed CAD based on radiomic features extracted from multimodal ultrasound images to classify breast tumors. The method used 181 breast tumors, of which 114 were benign and 67 were malignant. The noise is reduced using a directional filter bank, and segmentation is done using the contourlet transform method. Some of the shape features extracted from ROI include maximum-width-to-maximum-thickness ratio, solidity, convex area, orientation, long-axis length, short-axis length, perimeter, maximum width, area, equivalent diameter, eccentricity, mean, median, maximum thickness mean, and median. An SVM classifier is used to classify ultrasound images as benign versus malignant tumors. The method yielded 84.12% of accuracy, 78.80% of specificity, and 92.86% of sensitivity, and AUC was observed to be 91.90%.

Hsu et al. [48] proposed the CAD for breast tumor classification using quantitative features extracted from ultrasound parametric images. The proposed CAD used a total number of 160 ultrasound images of which 80 were benign and 80 malignant lesions. These images were acquired from the Kuang Tien General Hospital. The method used morphological and texture analysis based on the Nakagami parametric imaging. The morphological features extracted include solidity, roundness, extent, a short distance of standard deviation, tumor circularity, and long-axis-to-short-axis ratio. Three classifiers, fuzzy *c*-means, LR, and SVM, were used. The logistic regression outperformed the other two classifiers, with an accuracy of 89.40%, 86.30% specificity, and 92.50% sensitivity, and they reported the AUC curve to be 96.00%.

Chang et al. [49] proposed an XGBoost classifier for breast cancer classification. A total of 2964 breast cancer samples were collected from the Chung Shan Medical University Hospital, Jen-Ai Hospital, and Far Eastern Memorial Hospital. The results revealed that the single XGBoost method had a very high testing accuracy of 94.00%.

Gómez-Flores and Hernández-López [50] proposed a CAD which helped radiologists to classify breast cancer. The proposed CAD is based on 39 morphological features which describe breast tumor shapes to distinguish whether they are benign or malignant tumors. A total number of 2054 breast ultrasound images acquired from the National Cancer Institute (INCa) of Rio de Janeiro, Brazi, and 892 mammogram images acquired from the Breast Cancer Digital Repository (BCDR) were used for training. Morphological features were extracted based on region descriptors and boundary descriptors. The features extracted include elongatedness, form factor, solidity, normalized residual, value, convexity, roundness, elliptic-normalized skeleton, circularity, compactness, eccentricity, long-to-short-axis ratio, area difference with an equivalent ellipse, and elliptic-normalized circumference. The AUC was reported to be 82.0% in both databases.

Liu et al. [51] developed the CAD for breast tumor classification based on edge feature extraction. The morphological features were extracted from the ROI, which included roughness, regularity, aspect ratio, ellipticity, and roundness. The SVM classifier was adopted to classify images, whether they are benign or malignant lesions. The proposed method used a total number of 192 ultrasound images, including 71 malignant and 121 benign. The proposed method achieved 67.31% accuracy, 47.62% sensitivity, 80.65% specificity, 62.50% PPV, and 69.44% NPV.

Irfan et al. [52] proposed deep learning segmentation of ultrasonic breast lesion images by combined a dilated semantic segmentation network (Di-CNN) and morphological erosion operation. The segmented images were fed into DenseNet201 with transfer learning for feature extraction. A total number of 780 breast tumor were used in the study. The method employed CNN-activated feature vectors, DenseNet201-activated feature vectors, and support vector machine (SVM) to classify the breast tumor. The method achieved an accuracy of 90.11% based on CNN-activated feature vectors and accuracy of 98.45% based on DenseNet201-activated feature vectors combined with the SVM classifier and 98.9% precision.

Lahoura et al. [53] proposed a machine learning framework for cloud-based breast cancer classification using the extreme learning machine (ELM). The method employed AdaBoost, SVM, Naïve Bayesian, perceptron, and *k-*NN, and then, the ELM model was executed. The data used were from the Wisconsin Breast Cancer Diagnosis (WBCD). The dataset consisted of 569 entries and 32 attributes. The experimental results revealed that the method achieved an accuracy of 98.68%, recall of 91.30%, precision of 90.54%, and *F*_1_-score of 81.29%.

## 3. Applications of Machine Learning Model-Based HPO

In this section, an overview of ML is given, followed by a brief clarification of each algorithm used in this research.

### 3.1. Machine Learning (ML) Models

ML is a field of computer science that was founded in the late 1970s when it was very difficult to get the existing knowledge about artificial intelligence (AI) [54]. AI is aimed at analyzing hypotheses and designing computer systems that can perform tasks requiring biological attention to make decisions based on the completed task: for example, image recognition and perception [55]. Two common types of ML methods are as follows:

- Supervised ML is when training functions have correctly labeled data, for classification problems where labels include discrete values and regression problems where labels include continuous values
- Unsupervised machine learning, where training functions are trained on an unlabeled dataset, can be used for clustering, dimensionality reduction, and outlier detection algorithms and requires no external understanding.

ML is popular because it is more efficient, timely, and less expensive than deep learning methods, and because it does not require powerful computing hardware, it can be deployed in low- and middle-income countries [56–58]. These ML have been used in various applications: CAD, image registration, image segmentation, image fusion, image search, and annotation developed [59, 60]. The features extracted are based on the ROI and not the whole image [61]. Some ML classifiers based on tree-structure that have been used for breast cancer classification and are appropriate for our study are given below. Tree-structure means the ML algorithms based on decision trees that use model decisions [62, 63].

#### 3.1.1. *k*-Nearest Neighbor (*k*-NN) Classifier

*k*-NN is a simple machine learning algorithm because the classifier is created by manipulating the distances between data points, and the *k*-NN in the training dataset belongs to the predictor class of each sample tested [62]. The *k*-NN is used to distinguish all similarities in its neighbors' majority votes as either Euclidean or Minkowski metric, and its function is shown below as the distance between two data points is calculated in a plane.

(1) $$\begin{array}{c} d\left(x,x^{\prime }\right)=\sqrt{\overset{k}{\underset{i=1}{\sum }}\;{\left(x_{i}-x_{j}^{\prime }\right)}^{2}}, \end{array}$$

where *d* is the distance function, *n* is the number of variables, and *x*_*i*_ and *x*_*j*_′ are the variables of vectors *x* and *x*′, respectively, in the two-dimensional vector space. The function to calculate the distance between two data points in a norm vector space is given below:

(2) $$\begin{array}{c} d\left(x,x^{\prime }\right)={\left(\overset{k}{\underset{i=1}{\sum }}\;{\left(\;|\;x_{i}-x\prime_{i}\;|\;\right)}^{e}\right)}^{1/e}. \end{array}$$

In the above equation, the value of *e* can be manipulated.

#### 3.1.2. Support Vector Machines (SVM) Classifier

SVM is a well-known supervised ML algorithm that has been used for classification and regression problems. When choosing the best separating hyperplane for linearly classifying data, SVM uses a hyperplane for two different classes [62, 64, 65]. It can delineate the data points from low to high geographical space [66, 67]. The hyperplane expression is given below:

(3) $$\begin{array}{c} f\left(x\right)=w^{T}x+b. \end{array}$$

Furthermore, SVM-RBF can be adopted if the data is not split linearly and depends on the distance from its initial point to another point. The function is given below:

(4) $$\begin{array}{c} k\left(x,x^{\prime }\right)=\exp\left(-\gamma \;|\;{\left.\left|x-x^{\prime }\right|\right|}^{2}\right). \end{array}$$

In this, *γ* is the kernel variable for each kernel function. These variables influence the performance of SVM, and the distance used in the initial space is found by the similarity of *x* and *x*′.

#### 3.1.3. Random Forest (RF) Classifier

The RF classifier is widely used to create many trees in a forest. RF accuracy depends on the number of trees created in the forest. Therefore, RF accuracy is influenced by the number of trees generated in the forest, since the more trees in the forest, the more the accuracy increases significantly, and vice versa. In addition, RF uses batching and randomization in the construction of each tree when creating a forest of trees [62, 64, 65]. It is prescribed in the Gini index function below.

(5) $$\begin{array}{c} =1-\overset{c}{\underset{i}{\sum }}\;=1{\left(p_{i}\right)}^{2}. \end{array}$$

The function determines the number of nodes on the DT branch, where *p*_*i*_ constitutes the comparative frequency of the class noticed and *c* is the number of classes in the dataset.

#### 3.1.4. Gradient Boosting (XGBoost) Machine

XGBoost is one of the deterministic machine learning algorithms that can be applied to regression and classification problems. The classifier is very popular because it is reliable, efficient, predictable, and comparatively slow in implementation [63, 68]. In this way, it adapts to many of Kaggle's challenges.

#### 3.1.5. LightGBM Classifier

The LightGBM is one of the varieties of gradient enhancement structures created in DT. This structure can improve the efficiency of the model and reduce memory usage when splitting trees with the leaf method. In addition, it is widely used in various tasks such as feature ranking and classification [69, 70].

### 3.2. Hyperparameter Optimization (HPO) in Machine Learning Models

To achieve optimal ML results, the selection and tuning of hyperparameters are the most important factors. Hyperparameters are parameters that have been tweaked to improve their performance or accuracy in machine learning. These parameters are of greater importance when studying, creating, and evaluating machine learning classifiers. In addition, HPO is a procedure used to find the optimal hyperparameters in the ML classifier. Some of the HPO are default hyperparameters, hyperparameters based on genetic algorithms (GA), hyperparameters based on random search (RS), hyperparameters based on particle swarm optimization (PSO), hyperparameters based on hyperband, hyperparameters based on Bayesian optimization with Gaussian processes (BO-GP), and hyperparameters based on Bayesian optimization with tree-structured Parzen estimator (BO-TPE).

#### 3.2.1. Hyperparameter Based on Bayesian Optimization with Tree-Structured Parzen Estimator (BO-TPE)

BO-TPE is used to manage categorical, discrete, continuous, and conditional hyperparameters [62, 71, 72]. The tree-structured Parzen estimator (TPE) may be a sequential model-based optimization (SMBO) method. During this study, the BO-TRE was adopted since its performance is higher in some difficult problems, and it is also often better than other HPO [73, 74]. Moreover, it is ready to decide the most desirable hyperparameters or most close desirable hyperparameter configuration within a short time. TPE is one of the surrogate models for BO. Instead of defining a predictive distribution, the model generates two densities to act as generative models for all domain variables. In addition, the BO method has been used because it is more effective and can be used in many HPO problems. The model can perform better even if the objective function *f* is stochastic, nonconvex, or noncontinuous [62].

## 4. Materials and Method

This section gives an overview of the materials used and the method applied.

### 4.1. Acquisition and Segmentation

**Figure 1:**
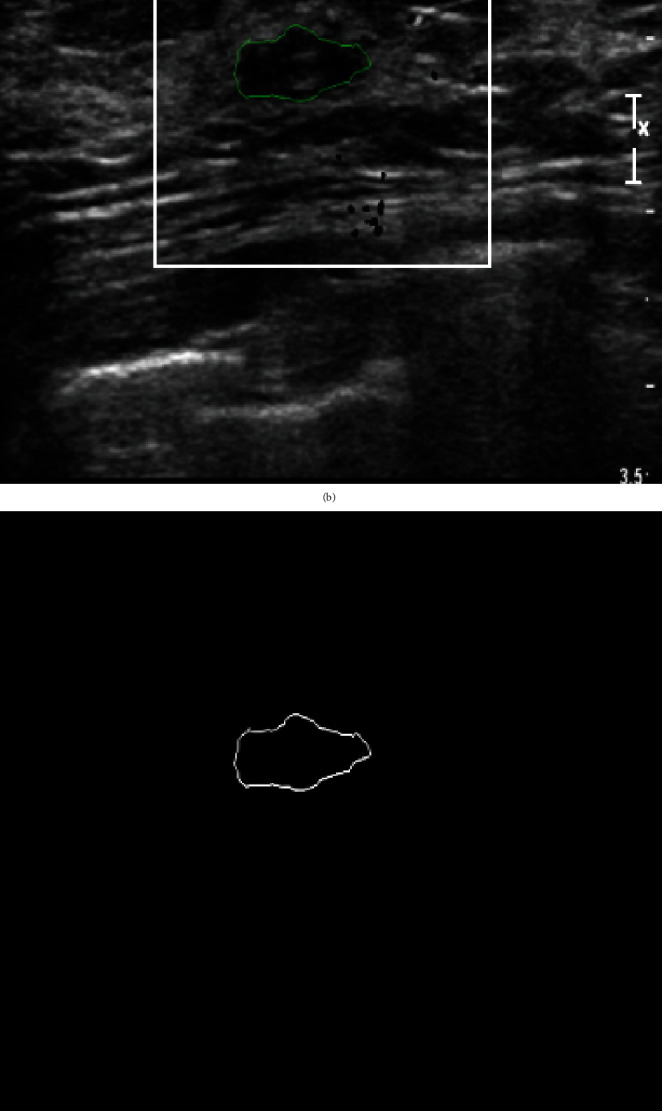
Benign: (a) original image, (b) mask image, (c) outline detection, and (c) ground truth image.

**Figure 2:**
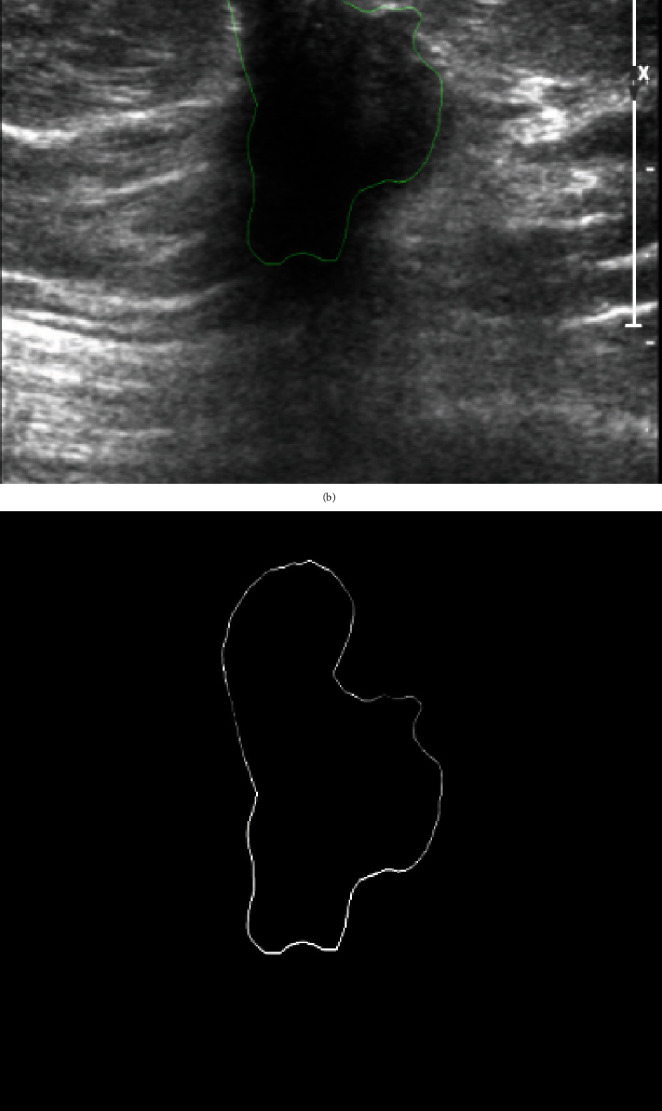
Malignant: (a) original image, (b) mask image, (c) outline detection, and (c) ground truth image.

The breast ultrasound images used in this dataset were obtained from a local hospital. The dataset contains 912 (512 × 512 with png extension) breast ultrasound images, including 600 benign and 312 malignant lesions confirmed by a pathology report, regardless of whether they are benign or malignant lesions. The images marked by medical radiologists with their corresponding label images are shown in Figures 1 and 2. Furthermore, based on a marked area, we have developed an algorithm for outline detection and segmentation based on binarization to obtain the ground truth. This was done with the help of OpenCV in the Python library. We developed an algorithm to detect the contour of an image, allowing us to identify the shape of images. The shape of images helps doctors determine whether they are normal, benign, or malignant. It detects the boundary of an image with the same intensity by joining all of the marks drawn by the physician. The image was thresholded to produce a binary image, and the flood fill from pixel (0, 0) was used to produce the inverted image. The inverted, flood-filled image aids in white-to-black and black-to-white conversion. The thresholded image is merged with the inverted flood-filled image bitwise to create the final foreground mask with holes filled in.

### 4.2. Feature Extraction

The dataset used in this paper contains 185 features extracted from 912 ultrasound images that belong to two classes, which are malignant and benign tumors, and the features are saved in tabular data with a CSV extension. Furthermore, the speckled noise was removed using a wavelet filter, which is a built-in filter in the PyRadiomics toolbox. The features were extracted using the PyRadiomics toolbox implemented in Python, which is an open-source package [75]. The setting parameters for the feature extraction were set to be minimumROIDimensions (2), minimumROISize (None), normalize (True), normalizeScale (256), removeOutliers (3), resampledPixelSpacing (None), interpolator (sitkBSpline), preCrop (False), padDistance (5), distances (1), force2D (True), force2Ddimension (0), resegmentRange (None), label (1), additionalInfo (True), binWidth (25), symmetricalGLCM (True), and weightingNorm (None).

### 4.3. Method

**Figure 3:**
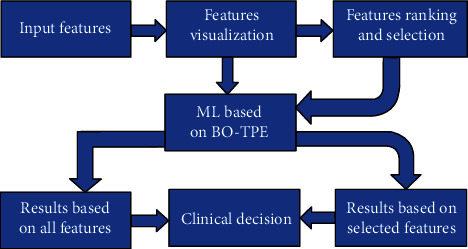
Proposed framework.

Five ML classifiers were used, including the support vector machine (SVM), *k*-nearest neighbor (*k*-NN), random forest (RF), XGBoost, and LightGBM. ML was optimized using the tree-structured Parzen estimator, and the dataset was divided using 10-fold cross-validation. The ML classifiers are used to classify features extracted from breast ultrasound images as benign lesions versus malignant lesions. The proposed framework is shown in Figure 3.

## 5. Experiment

This section introduced the setup of the experiment and results for the proposed five (5) ML models employed on 185 features extracted from 912 breast cancers. The dataset is split into two parts: 80 for training and 20 for testing. Thirteen features out of 185 features were used to train five ML models. The ML models were trained using 10-fold cross-validation and were optimized using BO-TPE. These features include the original mean (1); four (4) features extracted from 2D shapes such as elongation, major axis length, maximum diameter, and mesh surface; five (5) features extracted from first order shapes such as 90 percentile, median, minimum, minimum range, and maximum range; and three (3) features extracted from GLCM such as informational measure of correlation (IMC2) *L*, informational measure of correlation (IMC2) *H*, and maximum probability. Five machine learning classifiers were used in this study, which included *k*-NN, SVM, RF, XGBoost, and LightGBM, which were used to classify breast cancer. The performance of ML was measured using four metrics, including accuracy, precision, recall, and *F*-score. Each metric's detail and mathematical expression are provided below: Accuracy is defined as the ratio of correctly classified samples to total samples [76]. Its mathematical expression is shown below.

(6) $$\begin{array}{c} \text{Accuracy}=\frac{\text{TP}+\text{TF}}{\text{TP}+\text{TF}+\text{FP}+\text{FN}}. \end{array}$$

Precision can be defined as the ratio between true positive (TP) and the total of true positive (TP) and false positive (FP) [76]. Its mathematical expression is shown below.

(7) $$\begin{array}{c} \text{Precision}=\frac{\text{TP}}{\text{TP}+\text{FP}}. \end{array}$$

Recall can be defined as the ratio between true positive (TP) and the total of true positive (TP) and false negative (FN) [76]. Its mathematical expression is shown below.

(8) $$\begin{array}{c} \text{Recall}=\frac{\text{TP}}{\text{TP}+\text{FN}}. \end{array}$$

The *F*_1_-score is defined as the harmonic mean between precision and sensitivity [76]. Its mathematical expression is given below:

(9) $$\begin{array}{c} F_{1}-\text{score}=\frac{2\times \text{Precision}\times \text{Recall}}{\text{Precision}+\text{Recall}}=\frac{2\times \text{TP}}{2\times \text{TP}+\text{FP}+\text{FN}}. \end{array}$$

## 6. Experimental Results and Discussion

This section discusses experiment results, the importance of segmentation in practical solutions, and potential futures.

### 6.1. Results

**Figure 4:**
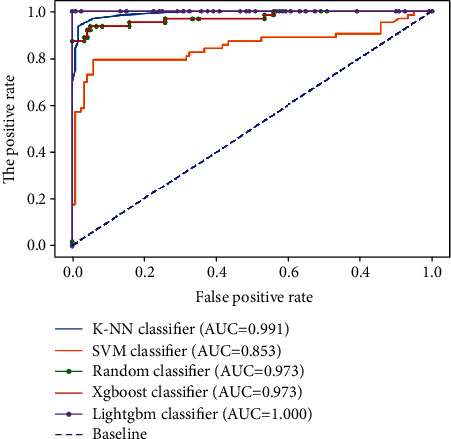
Performance comparison of five classifiers in terms of ROC.

This paper proposed a CAD approach that would help radiologists classify and detect breast cancer based on ultrasound images, whether they are benign or malignant lesions. Detecting whether breast lesions are benign or malignant with high accuracy and a low false rate is a significant step for breast cancer. The performance of the developed CAD was evaluated using accuracy, recall, precision, and *F*_1_-score. The features were ranked, and we selected only 13 out of 185 features. The feature selection is based on the embedded method. The feature selection is based on the embedded method. This method is used to combine the qualities of the filter and wrapper methods [77]. The embedded method belongs to decision tree algorithms that have their own built-in feature selection methods. According to Table 2, the performances of five classifiers were compared: *k*-NN, SVM, random, XGBoost, and LightGBM were used to classify the images. The region under the curve (ROC) of the proposed method is depicted in Figure 4. The LightGBM outperformed the other four classifiers as the accuracy, precision, recall, and *F*_1_-score which were noted to be 99.86%, 100.0%, 99.60%, and 99.80%, respectively. Because *k*-NN and SVM are simple models that are ineffective for high-dimensional datasets, they are underfitting.

### 6.2. Discussion

Breast cancer patients expect accurate results. Radiologists sometimes give inaccurate results when predicting that a patient has cancer when the patient may not actually have cancer. This scenario is possible due to the large number of ultrasound images generated each day and the limited number of radiologists who analyze them. This scenario automatically has implications for patients, if radiologists recommend that the patient has no cancer, while in the actual sense she has cancer. This will lead to unnecessary high costs in the future and sometimes death if the cancer is detected at a late stage. On the other hand, if radiologists recommend that a patient has breast cancer when, in reality, she does not have breast cancer, the patient might incur unnecessary costs as well as painful treatments due to the biopsy and stress.

### 6.3. The Importance of Segmentation in Practical Solutions

The segmentation method is applied in computer vision for the detection and identification of abnormalities in medical images [78]. Physicians can use the segmented area to assess tissue volume, diagnose diseases, locate pathology, examine anatomical structures, and plan for treatment. The segment part will help physicians draw conclusions about whether the segment part is normal or abnormal [79]. The segmentation is important because it helps physicians detect abnormalities and also diagnose diseases.

### 6.4. Potential Future

The proposed paper is based on classical methods for breast cancer classification. However, in our future work, we plan to employ deep learning methods for breast cancer. Deep learning methods need more data, so more data will be collected. In addition, a comparison between the swarm intelligence optimization algorithm and the BO-TPE optimization algorithm will be conducted.

## 7. Conclusion

The dataset used in this study includes 185 features extracted from 912 ultrasound images belonging to two classes of malignant and benign tumors. We saved the features as tabular data using the CSV extension. In addition, we introduced a CAD framework to help radiologists classify breast ultrasound images into benign and malignant tumors. Furthermore, we evaluated the proposed framework's performance using five (5) classifiers: *k*-NN, SVM, RF, XGBoost, and LightGBM. The experiment results revealed Bayesian optimization with a tree-structured Parzen estimator based on ML classifiers for 10-fold cross-validation; the LightGBM classifier outperformed the other four classifiers in accuracy, precision, recall, and *F*_1_-score, which were 99.86%, 100.00%, 99.60%, and 99.80%, respectively. Furthermore, we discovered that 86% of the work we reviewed relied on default hyperparameter values. The contribution of our research is as follows: the algorithm for outlier detection was developed, followed by feature extraction using the pyradiomics toolbox. Machine learning and hyperparameter optimization were discussed and summarized. The most effective classifier for clinical application was identified and recommended.

## Figures and Tables

**Table 1:** Summary of previous related state-of-the-art works.

Related works	Year	Technique	Database	Evaluation metric	HPO
[40]	2019	ANN	100	93.1% ACC (M), 90.4% (B)	Default
[41]	2019	SVM	82	94.12% ACC	Default
[42]	2019	SVM	323	95.98% ACC, 95.37% SEN, 97.29%, and SPE	Default
[43]	2019	SVM, Ada, LDA, and MLR	2032	89.0% ACC, 82.0% SEN, and 93.0% SPE	Grid search
[44]	2019	SVM	1061	75.94% ACC, 66.37% SEN, and 86.87% SPE	Default
[45]	2019	LDA	116	89.0% ACC	Default
[46]	2019	MNN	840	97.8% ACC	Default
[47]	2019	SVM	181	84.12% ACC, 92.86% SE, and 78.80% SPE	GA
[48]	2019	FCM, LR, and SVM	160	89.4% ACC, 86.3% SE, and 92.5% SP	Default
[49]	2019	XGBoost	2964	94.0% ACC	RS
[50]	2020	LDA	2054	82.0% AUC	Default
[51]	2020	SVM	192	67.31% ACC, 47.62% SEN, and 80.65% SPE	Default
[52]	2021	LDA	2054	82.0% AUC	Default
[53]	2021	SVM	192	67.31% ACC, 47.62% SEN, and 80.65% SPE	Default
Proposed	2021	LightGBM	912	99.86% ACC, 100.0% PE, 99.60% RE, and 99.80% *F*_1_	BO-TPE

**Table 2:** Performance comparison using 10-fold cross-validation.

Classifiers	Accuracy	Precision	Recall	*F*_1_-score	Parameters optimized
*k*-NN	92.99%	92.49%	91.47%	90.47%	*n*_neighbors = 17
SVM	96.17%	95.64%	96.56%	95.13%	*C* = 32.35
Random	95.08%	95.25%	94.69%	93.48%	max_depth = 45, *n*_estimators = 420, min_samples_split = 8, min_samples_leaf = 11
XGBoost	94.96%	95.08%	95.00%	93.41%	max_depth = 24, learning_rate = 0.256, *n*_estimators = 200
LightGBM	99.86%	100.00%	99.60%	99.80%	max_depth = 13, learning_rate = 0.123, *n*_estimators = 350, num_leaves = 6

## Data Availability

The datasets used in this study are available from the corresponding author on request.
